# WHF Recommendations for the Use of Echocardiography in Chagas Disease

**DOI:** 10.5334/gh.1207

**Published:** 2023-06-08

**Authors:** Kate Ralston, Ezequiel Zaidel, Harry Acquatella, Marcia Melo Barbosa, Jagat Narula, Yu Nakagama, Gustavo Restrepo Molina, Karen Sliwa, Jose Luis Zamorano, Fausto J. Pinto, Daniel Jose Piñeiro, Mariana Corneli

**Affiliations:** 1 World Heart Federation, Switzerland; 2 Sanatorio Güemes, Buenos Aires, Argentina; 3 Pharmacology Department, School of Medicine, University of Buenos Aires, Buenos Aires, Argentina; 4 Faculty of Medicine Universidad Central de Venezuela, Hospital Universitario and Centro Medico, Caracas, Venezuela; 5 Sócor hospital, Department of Echocardiography, Brazil; 6 Division of Cardiology, Mount Sinai St. Luke’s Hospital, New York, USA; 7 Dept of Parasitology, Osaka Metropolitan University, Japan; 8 School of Medicine, CES University, Medellín, Colombia; 9 Cape Heart Institute, Faculty of Health Sciences, University of Cape Town, South Africa; 10 CIBERCV, Ramón y Cajal University Hospital, Madrid, Spain; 11 Cardiology Department, Centro Hospitalar Universitário Lisboa Norte, CAML, CCUL, Faculdade de Medicina, Universidade de Lisboa, Portugal; 12 Faculty of Medicine, University of Buenos Aires, Buenos Aires, Argentina; 13 Instituto Cardiovascular de Buenos Aires, Argentina

**Keywords:** Chagas Disease, Echocardiography, cardiomyopathy, aneurysm, heart failure

## Abstract

Chagas disease (ChD) represents a significant health burden in endemic regions of Latin America and is increasingly being recognized as a global health issue. The cardiac involvement in ChD, known as Chagas cardiomyopathy (ChCM), is the most severe manifestation and a leading cause of heart failure and mortality in affected individuals. Echocardiography, a non-invasive imaging modality, plays a crucial role in the diagnosis, monitoring, and risk stratification of ChCM.

This consensus recommendation aims to provide guidance on the appropriate use of echocardiography in ChD. An international panel of experts, including cardiologists, infectious disease specialists, and echocardiography specialists, convened to review the available evidence and provide practical recommendations based on their collective expertise.

The consensus addresses key aspects related to echocardiography in ChD, including its role in the initial evaluation, serial monitoring, and risk assessment of patients. It emphasizes the importance of standardized echocardiographic protocols, including the assessment of left ventricular function, chamber dimensions, wall motion abnormalities, valvular involvement, and the presence of ventricular aneurysm. Additionally, the consensus discusses the utility of advanced echocardiographic techniques, such as strain imaging and 3D echocardiography, in assessing myocardial mechanics and ventricular remodeling.

## 1. Background, Objectives, and Methodology

### 1.1. Background and objectives

Chagas disease (ChD) is a neglected tropical disease caused by the protozoan *Trypanosoma cruzi*, with some of the most serious manifestations affecting the cardiovascular system. It is a chronic, stigmatizing condition, associated with poverty and affecting close to six million people globally [1]. Although historically the disease was limited to endemic areas of Latin America, widespread migration has led to Chagas becoming a global disease. In addition to the morbidity and mortality associated with the disease, the social and economic burdens on individuals and society are substantial [1]. Often called the ‘silent killer’, ChD is characterized by a long, asymptomatic phase in affected individuals. Approximately 30% then go on to develop chronic Chagas cardiomyopathy (ChCM) characterized by serious cardiovascular complications such as stroke, rhythm disturbances, and severe heart failure [2].

While electrocardiography (ECG) is the chief diagnostic tool by which the diagnosis of ChCM is made, echocardiography plays a critical role in the evaluation of consequent structural heart disease, and provides important information about prognosis [3]. Unfortunately, access to echocardiography is not widespread, particularly in the rural/remote communities in which Chagas disease is prevalent, and it can be prohibitively expensive even in urban environments [4]. Access to high quality and timely echocardiography at the primary care level or near where patients reside, must become a priority for health systems that care for individuals with ChD, facilitating early diagnosis and management of those suffering from cardiomyopathy. These efforts will require financing, strengthening of infrastructure, human resource development, and national policies to establish evidence-based use of diagnostics to ensure the greatest impact on patient care [4].

The ‘World Heart Federation Recommendations for the Use of Echocardiography in Chagas disease’, an evidence-based consensus, is a practical and patient centered document, chiefly focused on limited-resource environments, and is created for all stakeholders to provide an understanding of the role and necessity of echocardiography in the evaluation and care of an individual with ChD.

### 1.2. Methodology

The World Heart Federation (WHF) appointed a writing group consisting of 13 specialists and imaging experts representing all global regions, and a reviewing group of nine. To ensure a best practice approach and a broad consensus, the document was developed through an extensive review of published guidelines and research papers. The results of the initial appropriateness scoring presented in tables throughout the document were discussed by the writing group and a final round of independent scoring (modified Delphi method) was accomplished [5,6]. The final document is aligned with the ‘WHF Inter-American Society of Cardiology (IASC) Roadmap on Chagas disease’ [1].

## 2. Introduction and Classification of Chagas Disease

### 2.1. Introduction

Chagas disease is a neglected public health problem. Transmission of the *Trypanosoma cruzi* protozoa chiefly occurs through skin inoculation from contaminated feces of *Reduviidae* insects, present in the houses of impoverished rural communities [7]. Vector control programs have substantially decreased the number of infected individuals from about 18 million in 1990 to about 6–7 million at present [8], but about 70 million people remain at risk of acquiring the disease. In recent decades the movement of people from rural to urban areas and from endemic to non-endemic countries [9,10] has led to the problem becoming a global concern. Other mechanisms of transmission include oral (through ingestion of contaminated foods), mother to child, blood transfusion, organ transplantation, and less commonly, laboratory accidents.

The pathogenesis of cardiac disease due to ChD is not clear, though multiple mechanisms are likely to play a role, including direct toxicity of the parasite on myocardial tissue, autoimmune phenomena, microvascular alterations, and autonomic denervation [11].

Chronic ChD diagnosis requires two or more positive serology tests, and subsequent clinical examination. The presence of ChCM is commonly detected by electrocardiogram (ECG) demonstrating a characteristic pattern of abnormalities and is essential for identifying patients with cardiac manifestations of the disease [7,8,12]. A normal ECG is associated with a benign prognosis.

A subject with positive serology and a normal ECG should have repeated evaluation every few years. In patients with positive serology and an abnormal ECG, or those who are symptomatic, additional tests such as an echocardiogram are indicated, but access is often limited. However, due to the potential severity of the cardiac complications, and with improving availability to non-invasive cardiovascular imaging techniques, the indication for their use in asymptomatic patients is now more pertinent [3].

### 2.2. Classification of Chagas disease

#### Acute disease

The initial infection in Chagas disease is most commonly asymptomatic, or with few, non-specific symptoms. In the case of vector-borne transmission, the onset usually follows a 1–2 week incubation period and lasts for a period of 6–8 weeks. Acute cardiac presentations during this phase are extremely rare, and manifest with signs and symptoms indistinguishable from myocarditis of other infectious etiologies [13]. A small minority of patients in the acute setting present with fulminant myocarditis, most commonly those who are immunosuppressed [14,15] or those infected due to oral transmission, with ingestion of a high amount of infected *Triatoma*.

Patients in the asymptomatic acute phase are rarely detected, and as such do not usually receive antiparasitic treatment, although treatment has been shown to be beneficial in this phase, especially in children. The effectiveness of treatment in the chronic form without cardiac manifestations, is still controversial, with some indirect evidence supporting the possibility of a reduction in progression to cardiac disease [1,16].

#### Chronic disease

a) Indeterminate form

Individuals with a serologic diagnosis of ChD without obvious cardiac or gastrointestinal manifestations are referred to as having the ‘indeterminate’ form of the disease [17]. However subclinical cardiac pathology has been identified by sophisticated echocardiographic and other advanced modalities, despite normal ECG findings. This group is better described as Chagas disease ‘without clinically detectable cardiac pathology’ [16,17]. This stage may persist for decades, while a subset of these patients will develop chronic cardiac disease (Chagas cardiomyopathy) or/and gastrointestinal manifestations.

**Figure 1:**
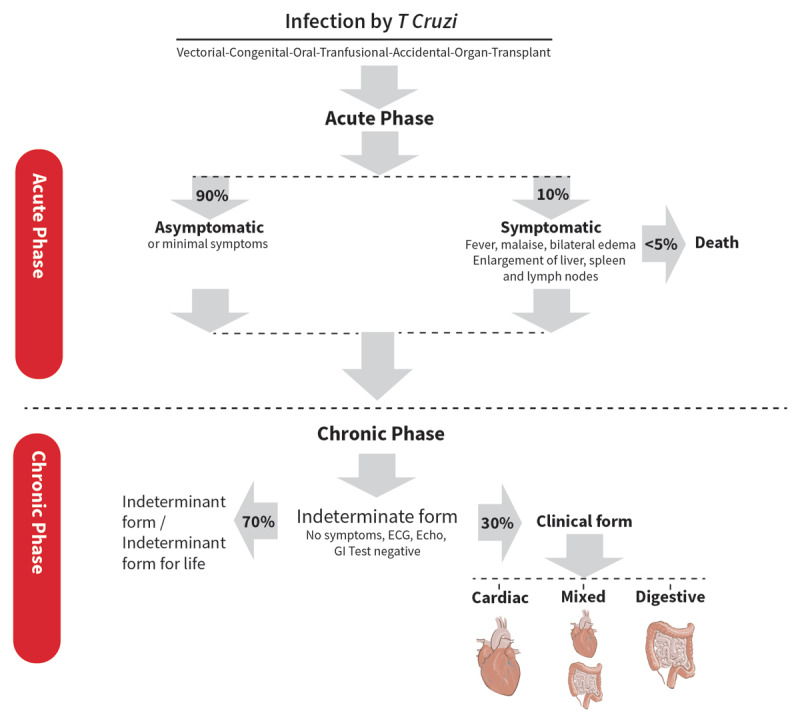
Phases of Chagas disease [1].

Following acute infection, in roughly a third of patients, the parasite evades the host immune defenses to establish the chronic forms of the disease. Orchestration of yet-to-be-defined parasite and host factors are thought to influence tissue tropism, and to be responsible for determining the magnitude of cardiac and/or gastrointestinal involvement in chronic ChD [12]. During this phase, effective antiparasitic therapy may result in parasitological cure and a reduction in progression to overt cardiac manifestations (Figure 1).

b) Chagas cardiomyopathy

Lacking effective treatment, in a subset of the population, Chagas cardiomyopathy will develop. It is progressive and malignant, affecting the heart, with the most common manifestations being heart failure, arrhythmias, heart block, thromboemboli, stroke, and sudden death [1,8,12,18].

## 3. Echocardiography

### 3.1. General contributions

Echocardiography plays a central role in the evaluation of ChD given its ability to detect cardiac damage and provide prognostic information. Impaired systolic function and increased ventricular dimensions are the most valuable findings in predicting cardiac morbidity and mortality [8,18,19,20,21,22,23].

In a patient with suspected ChD, it is advisable to perform a comprehensive transthoracic echocardiography (TTE), as described in the American Society of Echocardiography (ASE) recommendations for cardiac chamber quantification [24].

Portable ultrasound machines used to perform echocardiography may be an ideal tool in rural areas without easy access to facilities where a cardiology laboratory is readily available, [25,26] although clinical studies are still lacking on the use of portable echo for diagnosing patients with suspected ChD or with already established disease. A subjective evaluation showing left ventricular dysfunction may be enough for the diagnosis of ChCM. Table 1 summarizes recommendations on indications for performing echocardiography in ChD.

**Table 1:** Recommendations on indications for performing echocardiography.


INDICATION	APPROPRIATE (A), INAPPROPRIATE (I) OR UNDETERMINED (U)

Perform an echocardiogram for the diagnosis of Chagas disease	I

Perform a transthoracic echocardiogram to assess cardiac involvement in a patient diagnosed with Chagas disease	A

In areas with limited accessibility to health centers, it would be useful to perform a handheld echocardiogram to initially assess cardiac involvement	U

In rural areas with limited access to health centers, a POCUS protocol could be proposed to assess wall motion disorders, the presence of ventricular aneurysms, and left ventricular ejection fraction in patients with Chagas disease to determine cardiac involvement	U


### 3.2. Echocardiography in acute Chagas disease

**Figure 2:**
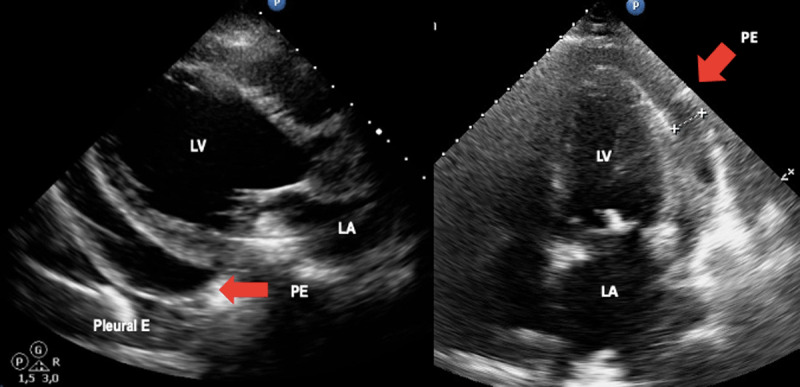
**Acute Chagas disease.** Arrows indicate pericardial effusion in heart failure due to Chagas disease. Image: Mariana Corneli. Reproduced with permission of the photographer.

In patients with clinically suspected acute ChD, echocardiography should be performed to assess for functional abnormalities, evaluate the presence of pericardial effusion, and to assess for the presence of acute myocarditis (Figure 2). In endemic countries or in patients from these countries presenting in non-endemic environments, a febrile illness accompanied by abnormalities in myocardial wall motion and/or pericardial effusion should raise the possibility of acute ChD in the differential diagnosis.

Global systolic function is rarely compromised, and wall motion abnormalities are usually segmental and focal [23]. In a series of 52 acute ChD myocarditis cases, the most common echocardiographic finding was pericardial effusion, which was detected in 42% (22 of 52) of the cases (mild to moderate in 17 and severe to massive in 5) [27]. Thus, among those with acute ChD who present with cardiomegaly on X-ray, echocardiography is more likely to detect pericardial effusion rather than chamber dilatation. In the case of a documented pericardial effusion, careful assessment for hemodynamic compromise should be performed. In Table 2 the stages of cardiac involvement in ChD are represented.

**Table 2:** Chagas disease staging.


ACUTE PHASE	CHRONIC PHASE				

	INDETERMINATE FORM	CHAGAS CARDIOMYOPATHY			

	A	B1	CHAGAS DILATED CARDIOMYOPATHY/HEART FAILURE		

			B2	C	D

Myocardial wall motion and/or pericardial effusion	Patients at risk for developing HF. They have positive serology, neither structural cardiomyopathy on ECG/echo nor HF symptoms.	Patients with structural cardiomyopathy, evident on ECG or echocardiographic abnormalities, but with normal or mildly depressed global LV function and neither current nor previous HF symptoms.	Patients with structural cardiomyopathy characterized by global LV dysfunction (LVEF > 40% < 50%) and neither current nor previous signs and symptoms of HF	Patients with ventricular dysfunction (LVEF < 40%) or V aneurism and current or previous symptoms of HF (NYHA FC I, II, III, or IV)	Patients with refractory symptoms of HF at rest despite optimized clinical treatment requiring specialized interventions


HF, heart failure; LV, left ventricular; LVEF, left ventricular ejection fraction; NYHA FC, New York Heart Association functional class. Adapted from Andrade JP et al. [6] and Nunes MCP et al. [7].

### 3.3. Echocardiography in chronic Chagas disease

#### 3.3.1 Left ventricle in ChCM

Two-dimensional echocardiography will determine left ventricular (LV) chamber size and detect diffuse hypocontractility or subtle regional wall motion abnormalities [18]. Left ventricular segmental abnormalities become more common over time, and are frequently located at the LV apex, inferobasal, and inferolateral walls, but may also affect other LV or right ventricular (RV) segments [28]. Regional wall motion abnormalities usually precede LV dysfunction and may be associated with ventricular arrhythmias even at early stages of the disease [29]. Even patients with indeterminate disease may have subtle changes in LV segmental contractility, detectable by speckle-tracking echocardiography [12,29].

The main pathologic finding in patients with ChD is a chronic, progressive myocarditis with fibrosis, and focal myocarditis may be found in patients with the indeterminate form [30]. Fibrosis is typically predominant in the posterior and apical regions of the LV, with involvement of the sinus node and electric conduction system, distinguishing ChD from other cardiomyopathies [31]. Assessment of myocardial strain through speckle-tracking echocardiography may allow early detection of subclinical myocardial dysfunction, in ChCM [12,30], as global longitudinal strain has a strong correlation with the amount of myocardial fibrosis as detected by cardiac magnetic resonance [32]. Regional strain is of particular significance, given the frequent segmental myocardial involvement described in apical, inferobasal, or inferolateral walls [32,33].

Speckle tracking echocardiography can also quantify the heterogeneity of systolic contraction, which is associated with the risk of arrhythmic events. A recent study showed that mechanical independent of LV ejection fraction [28,31,33].

Contrast echocardiography may be useful not only to better estimate LV systolic function and volumes, but also allows for the accurate detection of ventricular aneurysms and thrombi in ChD. With the apical four-chamber view, using contrast echo, it should be usually possible to clearly visualize the RV and LV cavities and detect segmental abnormalities [28].

ASE guidelines recommend two-dimensional echocardiography (2D echo) for the calculation of left ventricular ejection fraction (LVEF) using the biplane method of disks (the Simpson rule) [24]. Taking this into account, in patients with ChD it is recommended to evaluate systolic function using this methodology. However, the presence of apical LV aneurysms presents a challenge to the use of the method of disks [3]. Three-dimensional (3D echo) echocardiography allows visualization of cardiac chambers without geometric assumptions, and is more accurate than 2D echo for assessing LV volume and LVEF in patients with wall motion abnormalities, including small LV aneurysms, that would otherwise be overlooked by 2D echo because of foreshortening [24,34,35,36]. In these cases, calculation of LVEF using 3D echo would be beneficial, if available.

Echocardiography is also useful in evaluating dyssynchrony and in assessing a patient’s response to cardiac resynchronization therapy (CRT). Usual indications for CRT in these patients are when a pacemaker is needed in the presence of systolic dysfunction, in order to avoid further clinical worsening resulting from the dyssynchrony induced by artificial cardiac stimulation, or less frequently, when a patient has left bundle branch block (LBBB) and a low LVEF. However, there is little information regarding the benefit of CRT in ChCM. Although dyssynchrony may be present, most of the patients do not have LBBB, and there is little information regarding the benefit of CRT in ChCM [37,38]. However, there are some patients that receive a CRT when a replacement pacemaker is needed, or when a pacemaker is needed in the presence of systolic dysfunction, in order to avoid further clinical worsening resulting from the dyssynchrony induced by artificial cardiac stimulation, or less frequently, when a patient has LBBB a low LVEF.

Novel technologies like 3D echo and contrast echo, are not available in most of the places where ChD patients live. Table 3 summarizes recommendations on LV measurements.

**Table 3:** Recommendations for use of echocardiography in evaluation of the left ventricle.


PARAMETER OR INDICATION	APPROPRIATE (A), INAPPROPRIATE (I) OR UNDETERMINED (U)

2D Echo Assessment of LV Dimensions	A

Assessment by calculation of LVEF through the biplane method of disks (the Simpson rule)	A

LV evaluation in the presence of ventricular aneurysm by 3D echo if this methodology is available	A

Identify segmental wall motion disorders through 2D echo	A

Evaluation of LV systolic function by 2D echo in the presence of apical aneurysm and 3D echo available	I

Evaluation of global longitudinal deformity of the LV in the absence of motility disorders and preserved FEY in patients with suspected indeterminate form	A

It is recommended to evaluate the presence of ventricular dyssynchrony in patients with Chagas disease and impaired LVEF	A

Use of contrast echo in case of poor ultrasound window or in case of suspected left ventricular aneurysm if it is available	A

In a patient with Chagas disease and an echocardiogram without abnormalities, perform a stress echo with dobutamine to unmask ventricular dysfunction.	U


#### 3.3.2 Apical aneurysm and thrombi

**Figure 3:**
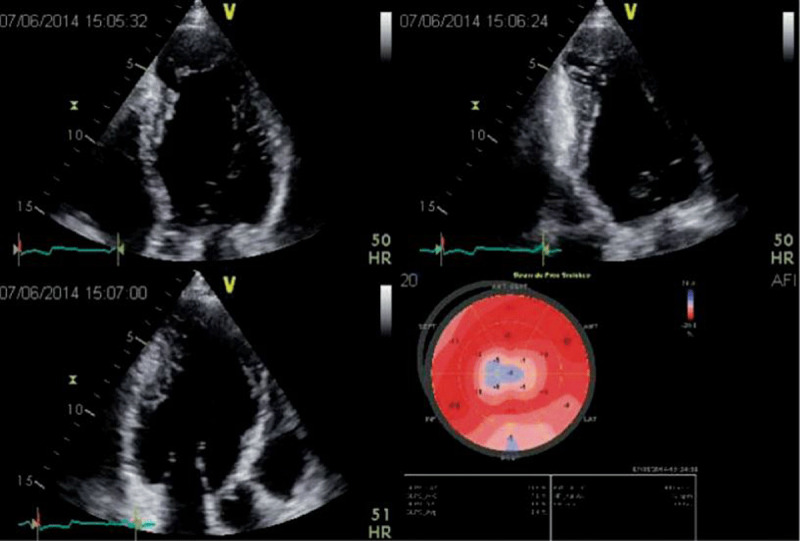
2D apical 4, 2 and 3-chamber views in a patient with Chagas disease and a typical aneurysm. In the 2-chamber view, a basal inferior aneurysm is also present. Longitudinal strain is abnormal in the apex, as well as on the basal inferior wall. Image: Marcia Barbosa. Reproduced with permission of the photographer.

Apical aneurysms are a hallmark of Chagas disease, so the presence of apical aneurysm should raise suspicion of ChCM [28,31,39]. These aneurysms are characterized using 2D echo as the presence of a deformity outside the endocardial border, persisting in systole and diastole. In general, these aneurysms are small, have a narrow neck, and may contain thrombi (Figure 3). Particular attention should be paid to spontaneous echo contrast and the presence of thrombus in ventricular chambers, especially associated with an aneurysm, as thrombi are important risk factors for systemic embolisms [40,41]. Importantly, these aneurysms are not always seen in the traditional apical views, and may be missed if a comprehensive echo is not performed. Unconventional views, especially a modified apical 2-chamber view, with posterior angulation of the transducer, are necessary to visualize the apical aneurysm and its associated thrombus.

The prevalence of aneurysms varies widely from study to study because of the heterogeneity of the population analyzed and the accuracy of the imaging method used for diagnosis. Nevertheless, LV apical abnormalities have a low prevalence in those with normal ECG findings but increase to 24% in those with an abnormal ECG [3,22].

#### 3.3.3 Left ventricular diastolic function

Similar to other cardiomyopathies, ChD may lead to impaired diastolic function, which can occur in the early stages of the disease. Diastolic dysfunction (DD) is an important diagnostic and prognostic factor in ChCM, and usually precedes systolic dysfunction. Accumulation of interstitial collagen fibers in the chronic form can lead to altered ventricular relaxation, followed by progressive reduced myocardial compliance, which then leads to increase LV filling pressure [42,43,44].

Thus, the first abnormality observed is abnormal LV relaxation, expressed as a decreased early mitral wave/late atrial wave (E/A) ratio, with prolongation of the isovolumic relaxation time and increased deceleration time of the E wave of the mitral valve. As the disease progresses, there is decreased LV compliance with secondary increased left ventricular filling pressures. This will increase the E/A ratio and decrease the relaxation time, with increased early mitral E wave/mitral annular early diastolic velocity by tissue Dopper (E/e’) ratio. Abnormalities of diastolic function have been described in all forms of ChD, increasing from stage A to more advanced stages of heart disease, with severe systolic and diastolic dysfunction.

The incidence of DD varies according to the stage of disease, ranging from about 10% in the indeterminate form to being almost universally present in ChD with heart failure [36]. In contrast, Pazin-Filho et al. looked at patients with the indeterminate disease, without echocardiographic markers of regional systolic dysfunction, and did not find any impairment of diastolic function [45]. Similarly, Barbosa et al. did not find any abnormality in diastolic function in patients with indeterminate stage disease, despite observed differences in regional LV contractility by two-dimensional speckle tracking strain [46]. The different findings in these studies are probably related to methodological differences in patient populations, selection of controls, and varied definitions of parameters for DD.

Doppler echocardiographic parameters to assess left ventricular filling pressures have been reported to enhance risk stratification in patients with impaired LV systolic function. The E/e’ ratio has provided the ability to determine the extent of diastolic dysfunction in several cardiac diseases. The inclusion of the E/e’ ratio in patients with ChCM has improved the risk prediction model beyond established risk factors, including functional class, LVEF, and RV function [47].

However, E/e’ ratio appears to have a different effect on mortality in the setting of ChCM, according to the degree of systolic dysfunction. Nunes et al. showed that in mild or moderate systolic dysfunction, an E/e’ ratio > 15 was a powerful predictor of mortality. In contrast, in severe systolic dysfunction, an increased E/e’ ratio was inversely associated with mortality which distinguishes ChD from heart failure of other etiologies [48]. The underlying mechanism to explain these findings is not known. Table 4 describes recommendations regarding the assessment of diastolic function in ChD.

**Table 4:** Recommendations for use of echocardiography in evaluation of left ventricle diastolic function.


PARAMETER OR INDICATION	APPROPRIATE (A), INAPPROPRIATE (I) OR UNDETERMINED (U)

Evaluate diastolic function parameters in all patients with suspected ChD or ChCM	A

Index left atrial volume in all patients evaluated with color Doppler echocardiography	A


#### 3.3.4 Left atrium

Diastolic dysfunction contributes to left atrium (LA) remodeling and dysfunction, particularly in patients with LV dysfunction [8,36,49]. Measurement of LA volume (LAV) and indexing it to body surface area (LAVi) has been demonstrated to be a marker of duration and severity of diastolic dysfunction. It is also useful in Chagas disease patients to predict prognosis. Left atrium volume can be increased in all stages of ChD [36,49,50], and LA function seems to be more significantly compromised in ChD than in idiopathic cardiomyopathy [51], suggesting a component of atrial myopathy. However, a study using strain to analyze atrial function demonstrated impairment to a degree similar to that of idiopathic dilated cardiomyopathy [52].

Left atrium volume indexed to body surface was an independent predictor of survival in a study of 192 patients followed for 34 months, adding incremental prognostic value to clinical factors, LVEF, and Doppler-derived parameters of diastolic function [50]. LA contractile function has also been shown as an independent predictor of subsequent clinical events [36]. Although LA electrophysiological properties were depressed in all groups of patients with the cardiac form, LA contractile function was decreased only in those with heart failure.

The importance of the evaluation of LV diastolic function and LA function in ChD is that end-systolic LV diameter, e’ velocity, and LA strain were associated with clinical events [36]. However, their clinical significance and role in staging of the disease remains to be determined.

#### 3.3.5 Right ventricle (RV)

Right ventricle systolic dysfunction may be an early finding in the natural history of ChD and has been detected in patients with the indeterminate and digestive forms [3,42,53,54]. It remains to be clarified whether RV dysfunction is predominantly secondary to chronic pulmonary hypertension induced by LV systolic dysfunction or reflects direct myocardial damage. Nevertheless, isolated right-sided heart failure is not frequent, and usually RV dysfunction is associated with LV dysfunction at advanced stages of ChCM [55].

Furthermore, it is important to emphasize that RV dysfunction may occur without symptoms or signs of heart failure, but may be aggravated by the burden generated by chronic pulmonary hypertension secondary to LV systolic dysfunction. In such circumstances RV dysfunction carries an adverse prognosis [56].

Evaluation of RV anatomy and function with echocardiography is often challenging [57]. Given the anatomical complexity of the RV, the evaluation of multiple echocardiographic parameters are suggested to determine RV compromise [24].

Assessment of regional wall motion abnormalities is difficult, mainly due to the presence of protruding trabeculae and the moderator band. Those structures cause suboptimal visualization of the wall motion of mid and apical RV segments. In the face of these limitations, echocardiographic evaluation of RV systolic function is usually performed using quantitative non-volumetric parameters. The tricuspid annular plane systolic excursion (TAPSE) is measured at the base of the RV free wall. Since the basal region of the RV is usually spared in ChCM, this might explain, at least in part, the lack of association between TAPSE and RV dysfunction.

Several indexes and methods have been used to describe RV dysfunction in patients with ChD showing somewhat discrepant results. For example, the myocardial performance index of the RV, also known as Tei index, provides incremental prognostic information about mortality, but could not identify RV involvement in the indeterminate form group of patients [24,46,56]. These mixed data may be attributed to the different methods used to assess RV function, as well as to the composition of the various groups of patients included in each study [42].

Given these limitations, novel technologies like 3D echo and myocardial strain through speckle-tracking echocardiography are being evaluated [46,57]. For example, analysis of RV deformation by using 2D speckle-tracking echocardiography improved detection of RV systolic dysfunction. However, the clinical value of these parameters needs to be better established in patients with ChD.

The presence of intracardiac devices such as pacemakers is common in patients with Chagas disease, for this reason it is important to evaluate in detail the location of the endocavitary catheters using all possible echocardiographic views. In this context, it is recommended to carefully evaluate the functional repercussions at the level of the tricuspid valve and its consequent degree of regurgitation.

Although the accuracy of RV volumes and RV ejection fraction is higher when using 3D echo, 2D echo has the advantage of ease of use and wider availability.

**Figure 4:**
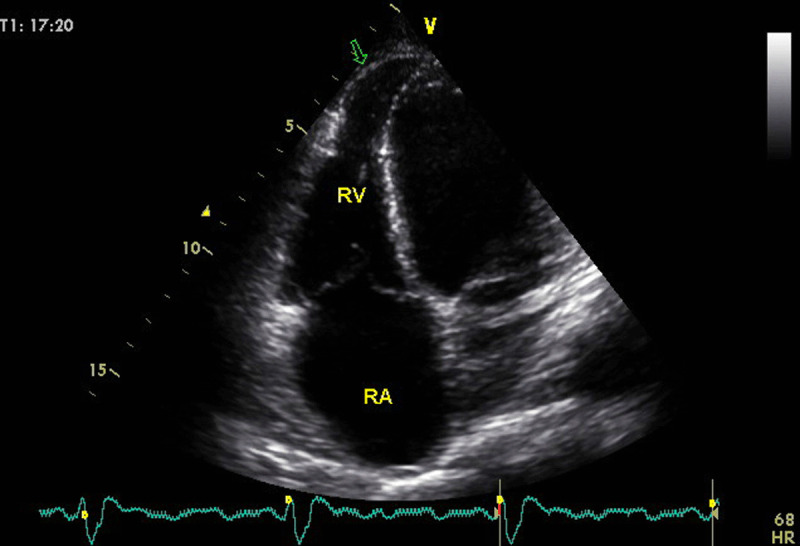
A young patient with Chagas disease presenting with biventricular dysfunction. Arrow in the 4 chamber-view, optimized to visualize the right ventricle, shows the infrequent finding of right ventricular aneurysm. Image: Marcia Barbosa. Reproduced with permission of the photographer.

Right ventricular aneurysms are uncommon, but some patients have apical aneurysms affecting both ventricles (Figure 4). It is important to check for the presence of thrombi in the RA and RV.

Right ventricular systolic involvement is a marker of ChD severity and represents a strong predictor of mortality [56,58]. Furthermore, it has also been reported to be an important determinant of exercise capacity in ChD. Right ventricular systolic annular velocity by tissue Doppler imaging was associated with peak VO2, regardless of the influence of age, gender, and LV systolic function [59]. Table 5 summarizes recommendations regarding RV echo measurements.

**Table 5:** Recommendations for use of echocardiography in evaluation of the right ventricle.


PARAMETER OR INDICATION	APPROPRIATE (A), INAPPROPRIATE (I) OR UNDETERMINED (U)

It is recommended to evaluate the dimensions and parameters of RV systolic function in all patients with Chagas disease when performing an echocardiogram.	A

Complement the evaluation of the right cavities with strain and 3D echo if these techniques are available	A

Estimate pulmonary systolic pressure in all patients with Chagas disease if possible	A

In the case of patients with Chagas disease and pacemakers or intracardiac devices, evaluate the functional repercussions at the level of the tricuspid valve and their adequate positioning.	A


#### 3.3.6 Valvular disease

Dilated cardiomyopathy is the most severe manifestation of ChD and can contribute to the presence of functional valve abnormalities such as mitral or tricuspid regurgitation (secondary regurgitation) [42].

In secondary mitral regurgitation (SMR), the valve leaflets and chordae are structurally normal and mitral regurgitation results from an imbalance between closing and tethering forces secondary to alterations in LV and LA geometry [60]. In ChD, severe LV dilatation has been associated with the presence of mitral valve regurgitation due to a dilatation of the mitral annulus. Also, in the case of wall motion disorders of the inferior and/or inferolateral segments, posterior leaflet tethering, despite almost normal LV size and ejection fraction, can be the mechanism, with a similar appearance to ‘ischemic MR’ [60].

In addition, ChD may also have a direct effect on the valves and be the direct cause of valvular heart disease (primary regurgitation). The pathogenesis of ChCM has not been completely elucidated, though the parasite-driven inflammatory reaction and the adverse host immune response are likely factors [61]. Resulting myocarditis, which is a feature of ChCM, frequently affects the papillary muscles leading to valve dysfunction, mainly regurgitation. This has been also reported as the main cause for the mitral valve prolapse seen in these patients [61].

The principal mechanism of tricuspid regurgitation in ChD is as a consequence of left-ventricular dysfunction. In some cases it may be secondary to pressure and/or volume overload mediated RV dilatation.

**Figure 5:**
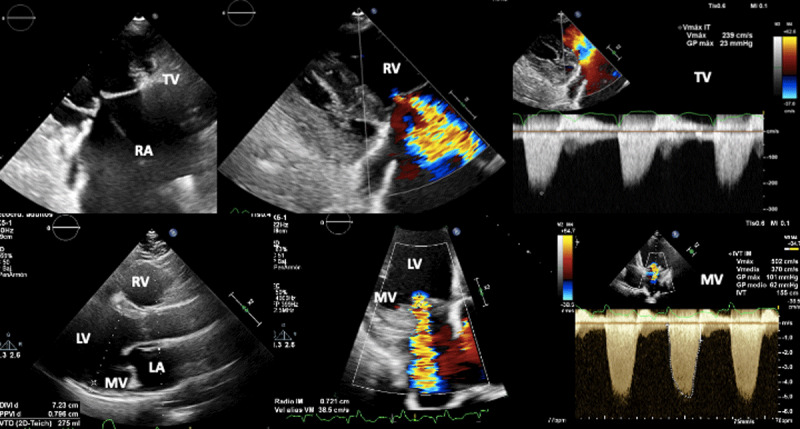
Biventricular dysfunction in a patient with Chagas disease. Superior figures show non coaptation of the tricuspid valve (due to right ventricular dilation) and its consequent functional regurgitation. Lower figures show mitral regurgitation secondary to left ventricular dysfunction. Image: Mariana Corneli. Reproduced with permission of the photographer.

The echocardiographic criteria to define severe mitral and tricuspid regurgitation do not differ from those used usually in both primary and secondary mitral and tricuspid regurgitation (Figure 5) (60).

Aortic valve pathology is less common, and potentially due to dilatation of the aortic annulus leading to aortic regurgitation.

## 4. Stages of CHCM

American Heart Association heart failure staging can be adapted to Chagas cardiomyopathy [8,18]. Asymptomatic patients with positive serology, who lack structural abnormalities evident on ECG or echocardiography are referred to as having ‘indeterminate’ ChD or ‘ChD without clinically detectable cardiac pathology’, and are referred to as having ‘Stage A’ disease.

Once cardiac impairment is evident on ECG or echocardiography, the patient is classified as ‘Stage B’ with the Chagas disease AHA scientific statement dividing this stage further in to B1 [asymptomatic ECG manifestations, wall motion abnormalities] and B2 [asymptomatic global LV dysfunction] [8].

Development of heart failure symptoms in a patient with structural cardiac changes identified by echocardiography is the hallmark of ‘Stage C’ illness. Finally, ‘Stage D’ chronic ChD patients are those at NYHA functional class IV with the clinical picture of decompensated, advanced heart failure generally associated with diffuse biventricular dysfunction and chambers dilatation [8,18,62].

Detection of cardiac involvement in the early stages of disease is particularly challenging, but of importance in determining requirements for follow up and risk stratification. Patients with true stage A disease need much less regular follow up, while those with incipient disease need more regular monitoring. Decline in ventricular systolic function parallels the staging of cardiac impairment and serves as the strongest predictor of survival in chronic ChD [19,63]. However, sudden cardiac death and ventricular arrhythmias may develop even with mild LV dysfunction [64].

## 5. When to perform an echocardiogram?

### 5.1. Indeterminate form (Stage A)

Asymptomatic Chagas patients with normal ECG and chest radiography: A normal ECG has strong negative predictive value for cardiovascular events and the majority of patients with a normal ECG will not develop cardiomyopathy. Those who do so, will have a 10 to 30 year lag period before worsening. A baseline echocardiogram is theoretically recommended [3,62] however, in considering the broader health system context in low resource settings, this recommendation should be qualified as non-urgent and should not be prioritized over other more pressing indications for echocardiography. Early detection of asymptomatic mild wall motion abnormality either by 2D echo echocardiography or, by strain does not infer a change in clinical management of this group of patients: they have the same prognosis as patients with normal echocardiogram and strain [8].

For subsequent, follow-up echo there is also no urgent requirement when a baseline echo is normal, while ECG and X-rays remain normal and the patient remains asymptomatic.

### 5.2. Chagas disease patients with ECG abnormalities (Stages B1 and B2)

Asymptomatic patients with ECG abnormalities: baseline echocardiography is recommended and the frequency of follow-up echo depends on the clinical status of the patient. Although studies on the progression of the disease are limited, it seems to be slow in asymptomatic patients with a normal echo [63]. Hence in these cases, repeat echo every five years is recommended, or when symptoms change. When the initial echo is abnormal, it should be repeated in one to three years, due to the increased risk of progression, especially if there is significant ventricular dysfunction. [3,62,65]. In any patient, the appearance of new symptoms or ECG abnormalities should be considered an indication for echocardiography.

The purpose of echocardiographic follow-up is to identify patients with moderate or severe LV dysfunction, who are often still asymptomatic but who could benefit from early administration of guideline-directed medical therapy [20,62,63,65]. Once disease stage B2 is established, periodic reassessment of patients is recommended to detect subtle signs of heart failure which may modify disease management. A periodic and specific assessment of LVEF deterioration, aneurysms and LV thrombi is mandatory.

### 5.3. Symptomatic patients (Stages C and D)

If significant LV dysfunction is present, recommendations for follow-up echocardiographic evaluation are similar to those for other forms of dilated cardiomyopathy. An echocardiogram should be repeated if there has been a deterioration in clinical status. An echocardiogram is also advised 3–6 months after optimization of standard therapies for heart failure with reduced ejection fraction (HFrEF) to determine the need for additional novel pharmacological agents like Angiotensin Receptor and Neprilysin Inhibitor Sacubitril-Valsartan or Sodium-Glucose Transporter 2 inhibitors dapagliflozin and empagliflozin and/or implanted devices [66].

Patients with symptomatic ChCM may present with diffuse hypokinesis. Nunes et al compared patients with idiopathic dilated cardiomyopathy versus patients with diffuse dilated ChCM, finding a worse prognosis in Chagas disease patients [32]. Similar results were found when comparing the survival of randomized patients in the PARADIGM-HF (Prospective Comparison of ARNI With ACEI to Determine Impact on Global Mortality and Morbidity in Heart Failure) and ATMOSPHERE (Aliskiren Trial to Minimize Outcomes in Patients With Heart Failure) trials [33]. For this reason, it is necessary to be familiar with this pattern of abnormalities that may point to ChD as the underlying etiology. Table 6 summarizes recommendations regarding when to perform an echocardiogram in patients with ChD.

**Table 6:** Recommendations on when to perform an echocardiogram in patients with Chagas disease.


SYMPTOMATIC STATE OF THE PATIENT	APPROPRIATE (A), INAPPROPRIATE (I) OR UNDETERMINED (U)

Asymptomatic patients with normal ECG should have a baseline echo, in a non-urgent fashion, where possible.	A

Asymptomatic patient with new changes in the ECG or patient that development symptoms that suggest cardiac compromise regardless of whether they have already undergone an echocardiogram previously	A

Asymptomatic patient without changes in the ECG with a previous echocardiogram performed less than 5 years ago without alterations	I

Repeat echocardiogram in patients with symptoms and impaired LV ejection fraction with impaired functional class	A

Repeat echocardiogram in symptomatic patients with LVEF <40% 3 to 6 months after adjusting medical treatment for heart failure to assess the impact of treatment	A

Repeat an echocardiogram annually in all patients with Chagas disease, regardless of the presence of electrocardiographic alterations or functional class, as an evolutionary control of the disease	I


## 6. Contributions of other cardiovascular imaging techniques

Cardiac magnetic resonance and radionuclide imaging provide complementary information to ECG and echocardiography in ChCM [42].

Using different cardiac magnetic resonance techniques and contrast images (late gadolinium enhancement), as well as radionuclide imaging with iodine-123-metaiodobenzylguanidine (^123^I-MIBG), SPECT, or PET with 18-Fluorodeoxyglucose, several findings and tissue characterization may be achieved, like microvascular disease, fibrosis-scars, edema, denervation [67,68,69,70,71,72,73,74,75,76]. These tests are not widely available but may further reclassify disease stages and progression rates.

## 7. Conclusions

Chagas disease is a serious public health concern in endemic countries and has become an emerging health problem in non-endemic areas due to migration and results in substantial morbidity and mortality. Echocardiography is a safe and cost-effective imaging tool to identify cardiac involvement in Chagas disease, offering the possibility of earlier diagnosis and prognostic information to improve management of patients. Thus, a comprehensive Doppler echocardiographic exam of patients with ECG abnormalities is fundamental, in order to detect ventricular abnormalities that may require treatment and offer prognostic information.

Despite the advantages of echo, it remains out of reach of many patients in the resource limited settings where the disease is most prevalent. Ironically it is in these settings that potentially the greatest benefits could be achieved in better managing patients at the point of care.

## Abbreviations

ASE: American Society of Echocardiography

BNP: brain natriuretic peptide

ChCM: Chagas cardiomyopathy

ChD: Chagas disease

DD: diastolic dysfunction

ECG: electrocardiogram

GRADE: Grading of Recommendations Assessment, Development, and Evaluation

LA: left atrium

LGE: late gadolinium enhancement

LV: left ventricular

LVEF: left ventricular ejection fraction

NSVT: non-sustained ventricular tachycardia

NYHA: New York Heart Association

POCUS: point of care ultrasound

RV: right ventricular

SMR: secondary mitral regurgitation

SCD: sudden cardiac death

TTE: transthoracic echocardiography

WHO: World Health Organization

## References

[1] Echeverría LE, Marcus R, Novick G, Sosa-Estani S, Ralston K, Zaidel EJ, et al. WHF IASC Roadmap on Chagas Disease. Glob Heart. 2020; 15(1): 26. DOI: 10.5334/gh.484 32489799 PMC7218776

[2] Ribeiro AL, Marcolino MS, Prineas RJ, Lima-Costa MF. Electrocardiographic abnormalities in elderly Chagas disease patients: 10-year follow-up of the Bambui Cohort Study of Aging. J Am Heart Assoc. 2014; 3(1): e000632. DOI: 10.1161/JAHA.113.000632 24510116 PMC3959704

[3] Acquatella H, Asch FM, Barbosa MM, Barros M, Bern C, Cavalcante JL, et al. Recommendations for Multimodality Cardiac Imaging in Patients with Chagas Disease: A Report from the American Society of Echocardiography in Collaboration With the InterAmerican Association of Echocardiography (ECOSIAC) and the Cardiovascular Imaging Department of the Brazilian Society of Cardiology (DIC-SBC). J Am Soc Echocardiogr. 2018; 31(1): 3–25. DOI: 10.1016/j.echo.2017.10.019 29306364

[4] Yadav H, Shah D, Sayed S, Horton S, Schroeder LF. Availability of essential diagnostics in ten low-income and middle-income countries: results from national health facility surveys. Lancet Glob Health. 2021; 9(11): e1553–e60. DOI: 10.1016/S2214-109X(21)00442-3 34626546 PMC8526361

[5] Norman D, Olaf H. An experimental application of the DELPHI method to the use of experts. Manag Sci. 1963; 9(3): 458–67. DOI: 10.1287/mnsc.9.3.458

[6] Patel MR, Spertus JA, Brindis RG, Hendel RC, Douglas PS, Peterson ED, et al. ACCF proposed method for evaluating the appropriateness of cardiovascular imaging. J Am Coll Cardiol. 2005; 46(8): 1606–13. DOI: 10.1016/j.jacc.2005.08.030 16226195

[7] Rassi A, Jr, Rassi A, Marin-Neto JA. Chagas disease. The Lancet. 2010; 375(9723): 1388–402. DOI: 10.1016/S0140-6736(10)60061-X 20399979

[8] Nunes MCP, Beaton A, Acquatella H, Bern C, Bolger AF, Echeverría LE, et al. Chagas Cardiomyopathy: An Update of Current Clinical Knowledge and Management: A Scientific Statement From the American Heart Association. Circulation. 2018; 138(12): e169–e209. DOI: 10.1161/CIR.0000000000000599 30354432

[9] Navarro M, Navaza B, Guionnet A, López-Vélez R. Chagas disease in Spain: need for further public health measures. PLoS Negl Trop Dis. 2012; 6(12): e1962. DOI: 10.1371/journal.pntd.0001962 23301105 PMC3531505

[10] Lima NA, Martin DT, de Castro RL, Ladzinski A, Ring A, Vos D, et al. Hospitalization for Chagas Heart Disease in the United States From 2002 to 2017. JAMA Netw Open. 2021; 4(10): e2129959. DOI: 10.1001/jamanetworkopen.2021.29959 34665243 PMC8527354

[11] Gascón J, Albajar P, Cañas E, Flores M, Gómez i Prat J, Herrera RN, et al. Diagnosis, management and treatment of chronic Chagas heart disease in areas where Trypanosoma cruzi infection is not endemic. Rev Esp Cardiol. 2007; 60(3): 285–93.DOI: 10.1157/13100280 17394874

[12] Rassi A, Rassi SG. Predictors of mortality in chronic Chagas disease: a systematic review of observational studies. Circulation. 2007; 115(9): 1101–8. DOI: 10.1161/CIRCULATIONAHA.106.627265 17339568

[13] Ortiz JV, Pereira BVM, Couceiro KDN, Silva MRHD, Doria SS, Silva PRLD, et al. Cardiac Evaluation in the Acute Phase of Chagas’ Disease with Post-Treatment Evolution in Patients Attended in the State of Amazonas, Brazil. Arq Bras Cardiol. 2019; 112(3): 240–6. DOI: 10.5935/abc.20190007 30916205 PMC6424035

[14] Gray EB, La Hoz RM, Green JS, Vikram HR, Benedict T, Rivera H, et al. Reactivation of Chagas disease among heart transplant recipients in the United States, 2012–2016. Transpl Infect Dis. 2018; 20(6): e12996. DOI: 10.1111/tid.12996 30204269 PMC6289649

[15] Bacal F, Silva CP, Pires PV, Mangini S, Fiorelli AI, Stolf NG and Bocchi EA. Transplantation for Chagas’ disease: an overview of immunosuppression and reactivation in the last two decades. Clin Transplant. 2010; 24(2): E29–E34. DOI: 10.1111/j.1399-0012.2009.01202.x 20088914

[16] Morillo CA, Waskin H, Sosa-Estani S, Del Carmen Bangher M, Cuneo C, Milesi R, et al. Benznidazole and Posaconazole in Eliminating Parasites in Asymptomatic T. Cruzi Carriers: The STOP-CHAGAS Trial. J Am Coll Cardiol. 2017; 69(8): 939–47. DOI: 10.1016/j.jacc.2016.12.023 28231946

[17] Chadalawada S, Sillau S, Archuleta S, Mundo W, Bandali M, Parra-Henao G, et al. Risk of Chronic Cardiomyopathy Among Patients With the Acute Phase or Indeterminate Form of Chagas Disease: A Systematic Review and Meta-analysis. JAMA Netw Open. 2020; 3(8): e2015072. DOI: 10.1001/jamanetworkopen.2020.15072 32865573 PMC7489816

[18] Andrade JP, Marin Neto JA, Paola AA, Vilas-Boas F, Oliveira GM, Bacal F, et al. I Latin American Guidelines for the diagnosis and treatment of Chagas’ heart disease: executive summary. Arq Bras Cardiol. 2011; 96(6): 434–42. DOI: 10.1590/S0066-782X2011000600002 21789345

[19] Tazar J, Arce Rojas N, Barbosa M. Alteraciones ecocardiográficas precoces en la enfermedad de Chagas crónica sin patología demostrada. Insuficiencia cardíaca. 2017; 12(3): 106–110.

[20] Yusuf S, Pitt B, Davis CE, Hood WB, Cohn JN, Investigators S. Effect of enalapril on mortality and the development of heart failure in asymptomatic patients with reduced left ventricular ejection fractions. N Engl J Med. 1992; 327(10): 685–91. DOI: 10.1056/NEJM199209033271003 1463530

[21] Rassi A, Little WC, Xavier SS, Rassi SG, Rassi AG, Rassi GG, et al. Development and validation of a risk score for predicting death in Chagas’ heart disease. N Engl J Med. 2006; 355(8): 799–808. DOI: 10.1056/NEJMoa053241 16928995

[22] Acquatella H. Echocardiography in Chagas heart disease. Circulation. 2007; 115(9): 1124–31. DOI: 10.1161/CIRCULATIONAHA.106.627323 17339570

[23] Fernandez AB, Nunes MC, Clark EH, Samuels A, Menacho S, Gomez J, et al. Electrocardiographic and echocardiographic abnormalities in Chagas disease: Findings in residents of rural Bolivian communities hyperendemic for Chagas disease. Glob Heart. 2015; 10(3): 159–66. DOI: 10.1016/j.gheart.2015.07.004 26407511 PMC4586045

[24] Lang RM, Badano LP, Mor-Avi V, Afilalo J, Armstrong A, Ernande L, et al. Recommendations for cardiac chamber quantification by echocardiography in adults: an update from the American Society of Echocardiography and the European Association of Cardiovascular Imaging. J Am Soc Echocardiogr. 2015; 28(1): 1–39.e14. DOI: 10.1016/j.echo.2014.10.003 25559473

[25] Chamsi-Pasha MA, Sengupta PP, Zoghbi WA. Handheld Echocardiography: Current State and Future Perspectives. Circulation. 2017; 136(22): 2178–88. DOI: 10.1161/CIRCULATIONAHA.117.026622 29180495

[26] Becker DM, Tafoya CA, Becker SL, Kruger GH, Tafoya MJ, Becker TK. The use of portable ultrasound devices in low- and middle-income countries: a systematic review of the literature. Trop Med Int Health. 2016; 21(3): 294–311. DOI: 10.1111/tmi.12657 26683523

[27] Parada H, Carrasco HA, Añez N, Fuenmayor C, Inglessis I. Cardiac involvement is a constant finding in acute Chagas‘ disease: a clinical, parasitological and histopathological study. Int J Cardiol. 1997; 60(1): 49–54. DOI: 10.1016/S0167-5273(97)02952-5 9209939

[28] Viotti RJ, Vigliano C, Laucella S, Lococo B, Petti M, Bertocchi G, et al. Value of echocardiography for diagnosis and prognosis of chronic Chagas disease cardiomyopathy without heart failure. Heart. 2004; 90(6): 655–60. DOI: 10.1136/hrt.2003.018960 15145872 PMC1768261

[29] Dias JC, Ramos AN, Gontijo ED, Luquetti A, Shikanai-Yasuda MA, Coura JR, et al. 2 nd Brazilian Consensus on Chagas Disease, 2015. Rev Soc Bras Med Trop. 2016; 49Suppl 1(Suppl 1): 3–60. DOI: 10.1590/0037-8682-0504-2016 27982292

[30] Rossi MA. Fibrosis and inflammatory cells in human chronic chagasic myocarditis: scanning electron microscopy and immunohistochemical observations. Int J Cardiol. 1998; 66(2): 183–94. DOI: 10.1016/S0167-5273(98)00208-3 9829333

[31] Barros, MV, Leren, IS, Edvardsen, T, Haugaa, KH, Carmo, AA, Lage, TA, et al. Mechanical Dispersion Assessed by Strain Echocardiography Is Associated with Malignant Arrhythmias in Chagas Cardiomyopathy. J Am Soc Echocardiogr. 2016; 29(4): 368–74. DOI: 10.1016/j.echo.2015.12.008 26833338

[32] García-Álvarez A, Sitges M, Regueiro A, Poyatos S, Jesus Pinazo M, Posada E, et al. Myocardial deformation analysis in Chagas heart disease with the use of speckle tracking echocardiography. J Card Fail. 2011; 17(12): 1028–34. DOI: 10.1016/j.cardfail.2011.08.007 22123367

[33] Gomes VA, Alves GF, Hadlich M, Azevedo CF, Pereira IM, Santos CR, et al. Analysis of Regional Left Ventricular Strain in Patients with Chagas Disease and Normal Left Ventricular Systolic Function. J Am Soc Echocardiogr. 2016; 29(7): 679–88. DOI: 10.1016/j.echo.2016.03.007 27086044

[34] Picard MH, Popp RL, Weyman AE. Assessment of left ventricular function by echocardiography: a technique in evolution. J Am Soc Echocardiogr. 2008; 21(1): 14–21. DOI: 10.1016/j.echo.2007.11.007 18165124

[35] Lang RM, Badano LP, Tsang W, Adams DH, Agricola E, Buck T, et al. EAE/ASE recommendations for image acquisition and display using three-dimensional echocardiography. J Am Soc Echocardiogr. 2012; 25(1): 3–46. DOI: 10.1016/j.echo.2011.11.010 22183020

[36] Nascimento CA, Gomes VA, Silva SK, Santos CR, Chambela MC, Madeira FS, et al. Left atrial and left ventricular diastolic function in chronic Chagas disease. J Am Soc Echocardiogr. 2013; 26(12): 1424–33. DOI: 10.1016/j.echo.2013.08.018 24055123

[37] Menezes Junior ADS, Lopes CC, Cavalcante PF, Martins E. Chronic Chagas Cardiomyopathy Patients and Resynchronization Therapy: a Survival Analysis. Braz J Cardiovasc Surg. 2018; 33(1): 82–8. DOI: 10.21470/1678-9741-2017-0134 29617506 PMC5873775

[38] Martinelli Filho M, de Lima Peixoto G, de Siqueira SF, Martins SA, Nishioka SA, Pedrosa AA, et al. A cohort study of cardiac resynchronization therapy in patients with chronic Chagas cardiomyopathy. Europace. 2018; 20(11): 1813–1818. DOI: 10.1093/europace/eux375 29509903

[39] Nunes MC, Dones W, Morillo CA, Encina JJ, Ribeiro AL, Cardiology CoCDotISo. Chagas disease: an overview of clinical and epidemiological aspects. J Am Coll Cardiol. 2013; 62(9): 767–76. DOI: 10.1016/j.jacc.2013.05.046 23770163

[40] Oliveira JS, Mello De Oliveira JA, Frederigue U, Lima Filho EC. Apical aneurysm of Chagas’s heart disease. Br Heart J. 1981; 46(4): 432–7. DOI: 10.1136/hrt.46.4.432 7295439 PMC482672

[41] Nunes MoC, Barbosa MM, Rocha MO. Peculiar aspects of cardiogenic embolism in patients with Chagas’ cardiomyopathy: a transthoracic and transesophageal echocardiographic study. J Am Soc Echocardiogr. 2005; 18(7): 761–7. DOI: 10.1016/j.echo.2005.01.026 16003275

[42] Nunes MCP, Badano LP, Marin-Neto JA, Edvardsen T, Fernández-Golfín C, Bucciarelli-Ducci C, et al. Multimodality imaging evaluation of Chagas disease: an expert consensus of Brazilian Cardiovascular Imaging Department (DIC) and the European Association of Cardiovascular Imaging (EACVI). Eur Heart J Cardiovasc Imaging. 2018; 19(4): 459–60n. DOI: 10.1093/ehjci/jex154 29029074

[43] Ribeiro AL, Nunes MP, Teixeira MM, Rocha MO. Diagnosis and management of Chagas disease and cardiomyopathy. Nat Rev Cardiol. 2012; 9(10): 576–89. DOI: 10.1038/nrcardio.2012.109 22847166

[44] Garcia-Alvarez A, Sitges M, Pinazo MJ, Regueiro-Cueva A, Posada E, Poyatos S, et al. Chagas cardiomyopathy: the potential of diastolic dysfunction and brain natriuretic peptide in the early identification of cardiac damage. PLoS Negl Trop Dis. 2010; 4(9): e826. DOI: 10.1371/journal.pntd.0000826 20877635 PMC2943653

[45] Pazin-Filho A, Romano MM, Gomes Furtado R, de Almeida Filho OC, Schmidt A, Marin-Neto JA, et al. Left ventricular global performance and diastolic function in indeterminate and cardiac forms of Chagas’ disease. J Am Soc Echocardiogr. 2007; 20(12): 1338–43. DOI: 10.1016/j.echo.2007.04.029 17764903

[46] Barbosa MM, Costa Rocha MO, Vidigal DF, Bicalho Carneiro ReC, Araújo RD, Palma MC, et al. Early detection of left ventricular contractility abnormalities by two-dimensional speckle tracking strain in Chagas’ disease. Echocardiography. 2014; 31(5): 623–30. DOI: 10.1111/echo.12426 25232573

[47] Nunes MC, Reis RC, Colosimo EA, Ribeiro AL, Barbosa FB, da Silva JL, et al. Risk estimation approach in Chagas disease is still needed. Int J Cardiol. 2011; 147(2): 294–6. DOI: 10.1016/j.ijcard.2010.12.044 21194765

[48] Nunes MP, Colosimo EA, Reis RC, Barbosa MM, da Silva JL, Barbosa F, et al. Different prognostic impact of the tissue Doppler-derived E/e’ ratio on mortality in Chagas cardiomyopathy patients with heart failure. J Heart Lung Transplant. 2012; 31(6): 634–41. DOI: 10.1016/j.healun.2012.01.865 22305956

[49] Fragata CaS, Matsumoto AY, Ramires FJ, Fernandes F, Buck PeC, Salemi VM, et al. Left Atrial Function in Patients with Chronic Chagasic Cardiomyopathy. Arq Bras Cardiol. 2015; 105(1): 28–36. DOI: 10.5935/abc.20150045 25993486 PMC4523285

[50] Nunes MC, Barbosa MM, Ribeiro AL, Colosimo EA, Rocha MO. Left atrial volume provides independent prognostic value in patients with Chagas cardiomyopathy. J Am Soc Echocardiogr. 2009; 22(1): 82–8. DOI: 10.1016/j.echo.2008.11.015 19131007

[51] Mancuso FJ, Almeida DR, Moisés VA, Oliveira WA, Mello ES, Poyares D, et al. Left atrial dysfunction in chagas cardiomyopathy is more severe than in idiopathic dilated cardiomyopathy: a study with real-time three-dimensional echocardiography. J Am Soc Echocardiogr. 2011; 24(5): 526–32. DOI: 10.1016/j.echo.2011.01.013 21353762

[52] Barbosa MM, Rocha MO, Botoni FA, Ribeiro AL, Nunes MC. Is atrial function in Chagas dilated cardiomyopathy more impaired than in idiopathic dilated cardiomyopathy? Eur J Echocardiogr. 2011; 12(9): 643–7. DOI: 10.1093/ejechocard/jer096 21771800

[53] Barros MV, Machado FS, Ribeiro AL, Da Costa Rocha MO. Detection of early right ventricular dysfunction in Chagas’ disease using Doppler tissue imaging. J Am Soc Echocardiogr. 2002; 15(10 Pt 2): 1197–201. DOI: 10.1067/mje.2002.122966 12411905

[54] Furtado RG, Frota DoC, Silva JB, Romano MM, Almeida Filho OC, Schmidt A, et al. Right ventricular Doppler echocardiographic study of indeterminate form of chagas disease. Arq Bras Cardiol. 2015; 104(3): 209–17. DOI: 10.5935/abc.20140197 25517391 PMC4386849

[55] Nunes MoC, Barbosa MeM, Brum VA, Rocha MO. Morphofunctional characteristics of the right ventricle in Chagas’ dilated cardiomyopathy. Int J Cardiol. 2004; 94(1): 79–85. DOI: 10.1016/j.ijcard.2003.05.003 14996479

[56] Nunes MoC, Rocha MO, Ribeiro AL, Colosimo EA, Rezende RA, Carmo GA, et al. Right ventricular dysfunction is an independent predictor of survival in patients with dilated chronic Chagas’ cardiomyopathy. Int J Cardiol. 2008; 127(3): 372–9. DOI: 10.1016/j.ijcard.2007.06.012 17689706

[57] Romano MMD, Moreira HT, Schmidt A, Maciel BC, Marin-Neto JA. Imaging Diagnosis of Right Ventricle Involvement in Chagas Cardiomyopathy. Biomed Res Int. 2017; 2017: 3820191. DOI: 10.1155/2017/3820191 28929112 PMC5592008

[58] Nunes MC, Carmo AA, Rocha MO, Ribeiro AL. Mortality prediction in Chagas heart disease. Expert Rev Cardiovasc Ther. 2012; 10(9): 1173–84. DOI: 10.1586/erc.12.111 23098153

[59] Nunes MoC, Beloti FR, Lima MM, Barbosa MM, Pinto Filho MM, de Barros MV, et al. Functional capacity and right ventricular function in patients with Chagas heart disease. Eur J Echocardiogr. 2010; 11(7): 590–5. DOI: 10.1093/ejechocard/jeq022 20304840

[60] Vahanian A, Beyersdorf F, Praz F, Milojevic M, Baldus S, Bauersachs J, et al. 2021 ESC/EACTS Guidelines for the management of valvular heart disease. Eur Heart J. 2022; 43(7): 561–632. DOI: 10.1093/eurheartj/ehab395 34453165

[61] de Almeida EA, Camargo LF, Lopes MA, de Castro Neto P, Salemi VM, Libermann A, et al. Mitral valve prolapse in chronic Chagas patients. Echocardiographic study. Arq Bras Cardiol. 1986; 47(3): 207–10. 3593020

[62] Ramos JM, Torrús D, Amador C, Jover F, Pérez-Chacón F, Ponce Y, et al. Multicenter epidemiological and clinical study on imported Chagas diseases in Alicante, Spain. Pathog Glob Health. 2012; 106(6): 340–5. DOI: 10.1179/2047773212Y.0000000039 23182138 PMC4005132

[63] Morillo CA, Marin-Neto JA, Avezum A, Sosa-Estani S, Rassi A, Rosas F, et al. Randomized Trial of Benznidazole for Chronic Chagas’ Cardiomyopathy. N Engl J Med. 2015; 373(14): 1295–306. DOI: 10.1056/NEJMoa1507574 26323937

[64] García-Chamorro LG, Zaidel EJ, Gheco L, Oliva MA, de-la-Vega A, Orosco A, et al. First appropriate implantable defibrillator shocks in patients with Chagasic heart disease. Arch Cardiol Mex. 2022; 92(3): 342–8. DOI: 10.24875/ACME.M22000326 34619749 PMC9262299

[65] Xavier SS, Sousa AS, Mallet AR, Hasslocher-Moreno A. Progressão da lesão miocardica na doença de Chagas: um estudo ecocardiográfico. Arq Bras Cardiol. 2004; 83(Suppl III): 127.

[66] McDonagh TA, Metra M, Adamo M, Gardner RS, Baumbach A, Böhm M, et al. 2021 ESC Guidelines for the diagnosis and treatment of acute and chronic heart failure. Eur Heart J. 2021; 42(36): 3599–726. DOI: 10.1093/eurheartj/ehab368 34447992

[67] Rochitte CE, Oliveira PF, Andrade JM, Ianni BM, Parga JR, Avila LF, et al. Myocardial delayed enhancement by magnetic resonance imaging in patients with Chagas’ disease: a marker of disease severity. J Am Coll Cardiol. 2005; 46(8): 1553–8. DOI: 10.1016/j.jacc.2005.06.067 16226184

[68] Romero J, Velasco A, Pisani CF, Alviz I, Briceno D, Díaz JC, et al. Advanced Therapies for Ventricular Arrhythmias in Patients With Chagasic Cardiomyopathy: JACC State-of-the-Art Review. J Am Coll Cardiol. 2021; 77(9): 1225–42. DOI: 10.1016/j.jacc.2020.12.056 33663741

[69] Mello RP, Szarf G, Schvartzman PR, Nakano EM, Espinosa MM, Szejnfeld D, et al. Delayed enhancement cardiac magnetic resonance imaging can identify the risk for ventricular tachycardia in chronic Chagas’ heart disease. Arq Bras Cardiol. 2012; 98(5): 421–30. DOI: 10.1590/S0066-782X2012005000031 22460166

[70] Senra T, Ianni BM, Costa ACP, Mady C, Martinelli-Filho M, Kalil-Filho R, et al. Long-Term Prognostic Value of Myocardial Fibrosis in Patients With Chagas Cardiomyopathy. J Am Coll Cardiol. 2018; 72(21): 2577–87. DOI: 10.1016/j.jacc.2018.08.2195 30466515

[71] Pacheco AB, Melo RJ, Rochitte CE. Cardiac Magnetic Resonance in the Assessment of Chagas Disease and its Complications. Int. J. Cardiovasc. Sci. 2020; 33(6): 705–12. DOI: 10.36660/ijcs.20200250

[72] Antonio M-NJ, Romano MMD, Maciel BC. Cardiac Imaging in Latin America: Chagas Heart Disease. CurrCardiovasc Imaging Rep. 2015; 8(9). DOI: 10.1007/s12410-015-9324-2

[73] Marin-Neto JA, Cunha-Neto E, Maciel BC, Simões MV. Pathogenesis of chronic Chagas heart disease. Circulation. 2007; 115(9): 1109–23. DOI: 10.1161/CIRCULATIONAHA.106.624296 17339569

[74] Miranda CH, Figueiredo AB, Maciel BC, Marin-Neto JA, Simões MV. Sustained ventricular tachycardia is associated with regional myocardial sympathetic denervation assessed with 123I-metaiodobenzylguanidine in chronic Chagas cardiomyopathy. J Nucl Med. 2011; 52(4): 504–10. DOI: 10.2967/jnumed.110.082032 21441532

[75] Lemos de Oliveira LF, Thackeray JT, Marin Neto JA, Dias Romano MM, Vieira de Carvalho EE, Mejia J, et al. Regional Myocardial Perfusion Disturbance in Experimental Chronic Chagas Cardiomyopathy. J Nucl Med. 2018; 59(9): 1430–6. DOI: 10.2967/jnumed.117.205450 29700129

[76] Simoes MV, Tanaka DM, Marin-Neto JA. Nuclear Medicine Methods for Assessment of Chronic Chagas Heart Disease. Int. J. Cardiovasc. Sci. 2020; 33(6): 686–9. DOI: 10.36660/ijcs.20200153

